# Investigation of a possible malaria epidemic in an illegal gold mine in French Guiana: an original approach in the remote Amazonian forest

**DOI:** 10.1186/s12936-019-2721-2

**Published:** 2019-03-22

**Authors:** Maylis Douine, Alice Sanna, Helene Hiwat, Sébastien Briolant, Mathieu Nacher, Didier Belleoud, François Michel Le Tourneau, Hervé Bogreau, Franck De Laval

**Affiliations:** 1 Centre d’Investigation Clinique Antilles-Guyane – Inserm1424, Cayenne Hospital, Cayenne, French Guiana; 2 Health Regional Agency, Cayenne, French Guiana; 3 National Malaria Programme, Ministry of Health, Paramaribo, Suriname; 4 French Armed Forces Institute for Biomedical Research (IRBA)/BAT/Parasitology and Entomology Unit, Marseille, France; 5 Epidemiology of Tropical Parasitoses, EA 3593, Université de Guyane, Cayenne, French Guiana; 6 French Armed Forces Health Service in French Guiana, Cayenne, French Guiana; 7 iGLOBES UMI 3157 Joint Research Unit, CNRS/University of Arizona, Tucson, USA; 8 French National Centre for Malaria (CNR Paludisme), Marseille, France; 9 IRD, AP-HM, IHU-Méditerranée Infection, UMR Vecteurs – Infections Tropicales et Méditerranéenne (VITROME), Aix Marseille Université, Marseille, France

**Keywords:** Malaria, Outbreak, Gold mining, Guiana Shield, Amazonia, Investigation

## Abstract

**Background:**

In April 2017, Suriname’s Ministry of Health alerted French Guiana’s Regional Health Agency (RHA) about an increase of imported malaria cases among people coming from an illegal gold mining site called *Sophie*, in French Guiana, a French overseas territory located in the Amazonian forest.

**Methods:**

Due to safety issues and the remoteness of *Sophie*, the RHA requested the collaboration of the French Armed Forces for the epidemiological investigation. A medical unit, and six soldiers to ensure the security of the mission, were transported by helicopter.

**Results:**

During the investigation, two malaria episodes were diagnosed among 46 persons. Twenty-six of them were from *Sophie*, where PCR-*Plasmodium* prevalence was estimated at 60% (15/26). This result was concordant with previous studies revealing high malaria endemicity in the gold miner population. The increase of imported cases in Suriname may have resulted from decreased access to under-the-counter anti-malarials and increased migration of gold miners to Suriname following a decline of the profitability of gold mining in a context of increased repression against illegal mining by the French army.

**Conclusion:**

This investigation of a suspicious malaria epidemic confirms the importance of malaria among illegal gold miners. Their mobility along the Guiana Shield and their health-seeking behaviour are likely to spread malaria in populations for which significant efforts are undertaken to fight against this disease. Fighting malaria in this population remains more relevant than ever. A pilot study (Malakit project) is currently in progress to evaluate the efficacy of kits for self-diagnosis and self-treatment.

## Background

French Guiana (FG) is a French overseas territory, thus a part of the European Union, located in South America. Apart from a narrow coastal strip, over 90% of the territory is covered by dense Amazonian rain forest. Malaria has been endemic in this region but, according to the epidemiologic surveillance system (Santé Publique France), incidence has been decreasing in the past 10 years, from 3265 cases in 2008 to 597 in 2017 [1]. Endemic species are *Plasmodium falciparum* and *Plasmodium vivax*, transmitted mainly by *Anopheles darlingi* [2].

However, undetected by the surveillance system (which is based on passive case detection at health care facilities), a silent but massive endemic transmission still occurs in illegal gold mining areas deep inside the forest [3, 4]. Between 2005 and 2015, illegal gold miners (called *garimpeiros*), who originate almost exclusively from Brazil, have been estimated to represent a population of 10,000 to 15,000 people. Although their exact number is not known, increased repression is estimated to have shrunk the size of this population to around 5000 in 2018, according to official sources. Garimpeiros travel between French Guiana and Brazil on the eastern side, crossing the Oiapoque River, and between French Guiana and Suriname on the western side, crossing the Maroni River. The alluvial gold extraction in placers upsets the river beds and the water streams, resulting in the formation of puddles of stagnant water which promote vector proliferation. Illegal immigration and activities, associated with isolation, hamper access to health care and lead to widespread gametocyte carriage which leads to sustained malaria transmission [3]. The abundance of vectors and parasites maintains endemic malaria.

Previous studies have shown a high prevalence of malaria among garimpeiros, up to 48.3% depending on mining sites [3, 4]. A large proportion of *Plasmodium* carriers were asymptomatic (48.5% to 84%), reflecting partial immunity consistent with intense malaria transmission. Their mobility, for logistical or family reasons, spreads malaria beyond borders in Suriname and Brazil, as reported by local health authorities [4–7]. Moreover, the behaviour of garimpeiros when they feel sick, relying on self-medication based on artemisinin derivatives (52%) and poor adherence (40%), may lead to the emergence and spread of artemisinin resistance in *P. falciparum* populations [4, 8, 9], with a risk aggravated by the circulation of counterfeit or expired medication at the gold mines [10, 11].

Because of policing operations in mining areas, despite doxycycline prophylaxis, military personnel are also very affected by malaria confirming high levels of malaria transmission. During the 2008–2017 period, 1070 malaria attacks were reported by the French Armed Forces’ epidemiologic surveillance system [12, 13].

### Outbreak detection

**Fig. 1:**
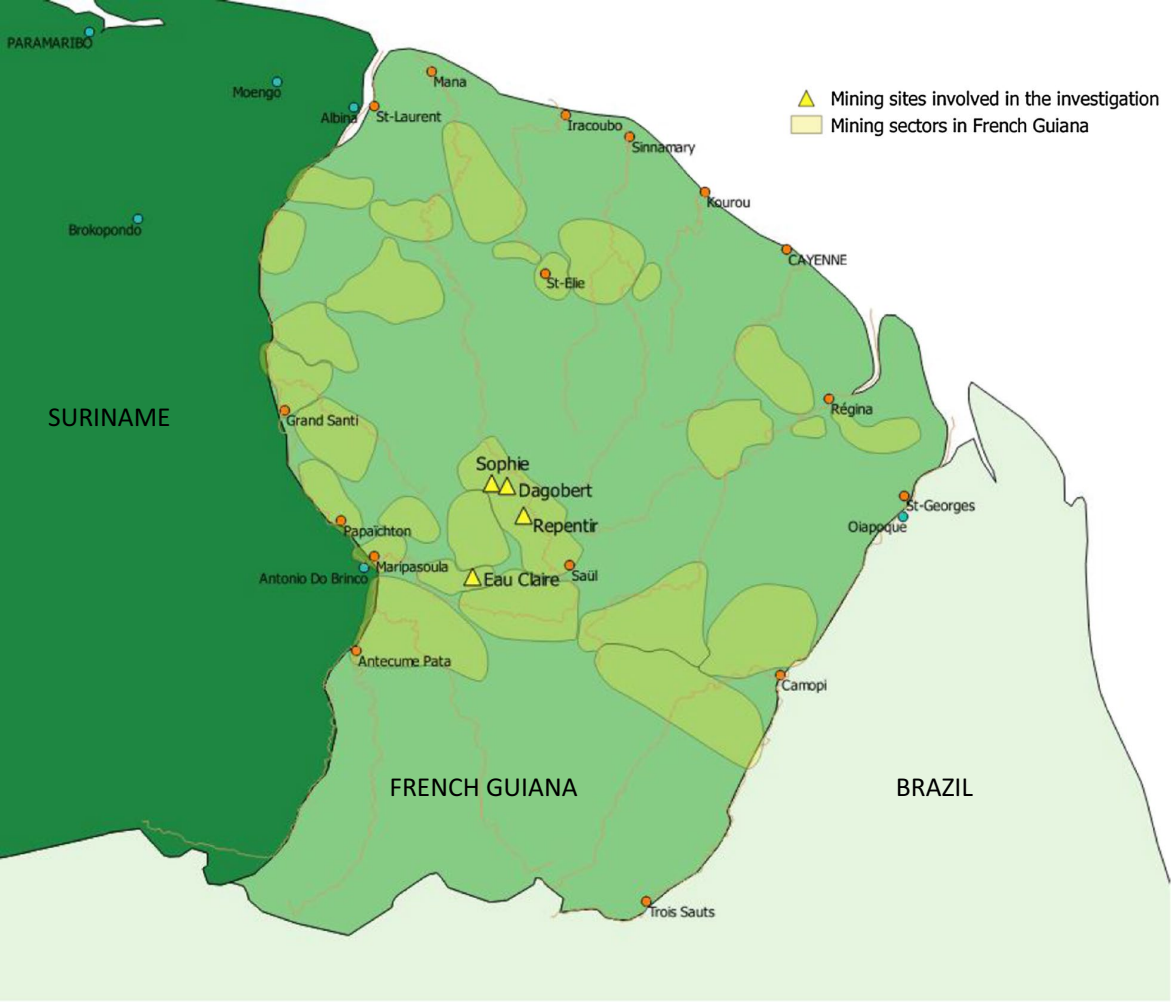
Localization of mining camp involved in this investigation, French Guiana

In April 2017, Suriname’s Ministry of Health alerted FG’s Regional Health Agency (RHA) that three Malaria Services Posts (Antonio do Brinco, Albina, and Paramaribo) notified 119 imported malaria cases from FG between January and April 2017, versus 45 during the same period in 2016. Sixty-three were *P. falciparum* (53%), 50 were *P. vivax* (42%), and 6 were mixed infections (5%). The declared place of contamination was *Sophie,* an illegal gold mine in FG (Fig. 1).

The signal, however, was not noted by Santé Publique France which relied on data coming from health facilities near *Sophie*, namely in Maripasoula, Saül and Mana, nor by the French military health service among soldiers returning from the surroundings of *Sophie*.

Several hypotheses were proposed to explain this signal. The first hypothesis was an increase in the number of garimpeiros working in FG, responsible for either a simple increase of the absolute number of malaria attacks, or a real increase of malaria incidence in newly arrived garimpeiros without any previously acquired immunity. The second hypothesis was an increase of gold miners moving to Suriname, either because of an easier access to care in Suriname, either because of an increase of military operations in FG. Finally, a decrease of the availability of under-the-counter malaria treatment in forest could also have explained the signal.

In order to validate the signal and to test these different hypotheses, an exploratory mission at the *Sophie* gold mining site was organized. The secondary objective of the mission was to ensure on site medical care, assess the situation, and to collect useful information for the preparation of further epidemic control missions, if the outbreak was confirmed.

## Methods

Illegal gold mines in the French Amazonian forest are generally located in remote isolated areas sometimes only accessible after several days of boat and walking, or by helicopter. Communications are scarce as there is no official infrastructure. Safety for medical personnel is not guaranteed since the garimpeiros operate in a clandestine environment. Thus implementing the explanatory mission raised several issues:

- to find a transport and a pilot who knows how to land inside the forest.
- to secure the perimeter to enable a safe investigation.
- to bring the investigation material but also the logistical support.
- to agree to a humanitarian truce without any police activity.

In this context the RHA requested the collaboration of the French Armed Forces in FG. A medical unit was transported by helicopter directly at the *Sophie* location. The team included two physicians with experience in outbreak investigation and field epidemiology (one from the Clinical Investigation Center–Cayenne’s Hospital, and one from the French Military Health Service), one health assistant fluent in Portuguese, and six soldiers to ensure the security of the mission. The capacity of the Puma helicopter was the main limiting factor, determining the trade-off between the ideal duration of the investigation, versus the weight of food and supplies. Shortening the mission to 5 days allowed to carry 20 kg of medical supplies.

The first day the team patrolled through the forest to search for shelter places, to explain the purpose of the mission, build trust, and choose the best place to settle. It was not possible to forecast whether garimpeiros would seek care at the medical unit, especially because of the military aspect of the mission. Because of these difficulties, two kinds of investigation were implemented.

### Case detection

A medical consultation was offered. Clinical examination looked specifically for malaria symptoms as fever, pallor, jaundice, and splenomegaly. A malaria rapid diagnostic test (RDT) (SD BIOLINE MALARIA PF/PAN^®^) was performed to any patient with malaria symptoms and/or temperature higher than 37.8 °C or declaring having fever in the past 24 h. RDT-positive persons were treated with artemether–lumefantrine for *P. falciparum,* or chloroquine for *P. vivax*, according to World Health Organization (WHO) guidelines [14].

In order to improve the evaluation of malaria prevalence, four blood drops were collected onto blotting paper (FTA^®^ card, Whatman^®^) from all patients encountered during the mission. Blotting papers were dried, packed and then sent to the National Reference Centre for Malaria, (Marseille, France), to perform *Plasmodium* detection by RT-PCR subsequently as it does not involve any specific care according to French Protocol. Parasitic genomic DNA was extracted from FTA^®^ card (Whatman^®^) using Magmax^®^ Robot (Life Technologies^®^) with the MagMAX™-96 DNA Multi-Sample Kit following supplier protocol (PN 4425070).

Malaria species diagnosis was based on four simple real time PCRs targeting four genes: aquaglyceroporin (access n°: AJ413249), enoyl-acyl carrier protein reductase (access n°: P. VIVAX_113890), circumsporozoite (accession number: S69014) and ookinete surface protein 25 (access n°: AB074976) in *P. falciparum, P. vivax, Plasmodium malariae* and *Plasmodium ovale,* respectively. Each singleplex real time PCR was carried out on LightCycler^®^ with the Roche LightCycler^®^ TaqMan Master kit. Cycling condition, common for all four species, consist of 95 °C (10 min) followed by 45 PCR cycles (95 °C [10 s], 60 °C [30 s]) and 40 °C (30″). Sequences of primers and probes are detailed in Table 1.

**Table 1:** Sequences of primers and probes for *Plasmodium* PCR

*Plasmodium* species	Genes	Acess number	Sens	Sequencies (5′ → 3′)
*Falciparum*	AQP	AJ413249	F	TTTATGTATTGGTATAACATTCGG
			R	GGCAAATAACTTTATCATAGAATTGAC
			P	TACACTACCAACACATGGGGCTACAAGAGGT
*Vivax*	EACPR	PVX_113890	F	GTGGCCGCCTTTTTGCT
			R	CCTCCCTGAAACAAGTCATCG
			P	CATCTACGTGGACAACGGGCTCAACA
*Malariae*	CS	S69014	F	GAGGAATGGTCACCATGTAGTGT
			R	CAAATTTCAGTTTCAAGGTCACTTAA
			P	ATTTTTTATCAACCTTTCTTCTAGCCC
*Ovale*	Pos25	AB074976	F	CCAAGCCCAGATAATAAGGAAGGT
			R	TTCGTGCACTTCAACTTACATTCAGT
			P	TTATTGTCCTCTGGGTTTGGAACTTTGCC

Clinical malaria was defined as evocative symptoms AND a positive malaria RDT. Asymptomatic malaria carriage (AMC) was defined as absence of evocative symptoms (regardless of splenomegaly) AND a positive RT-PCR-*Plasmodium* spp. Malaria parasite prevalence was defined by the number of *Plasmodium* positive RT-PCR divided by the number of persons tested.

The population size of gold miners in that mining camp was estimated by calculating the average of the estimations given by each patient. Historical incidence data did not exist, but two studies previously assessed malaria prevalence in garimpeiros [3, 4].

### Indirect approach by questionnaire

An anonymous semi-quantitative questionnaire was proposed to all volunteers collecting quantitative data (socio-demographic, mobility, medical history, personal malaria history, number of personal and witnessed malaria attacks during the last 3 months), and qualitative assessment of the following items: feeling that there is a current malaria outbreak, access to and behavior towards antimalarial treatment, modification of garimpeiros mobility during the last 3 months.

### Regulatory process

In the context of an outbreak investigation requested by the health authorities, no ethical committee validation was required. The data collected were anonymous. All patients willingly came to the medical consultation, and an informed consent was collected before sampling a blood drop, informing them that medical treatment was not tied to consent.

## Results

From June 15th to 20th 2017, 50 medical consultations were conducted, concerning 46 patients. Twenty-six came from *Sophie* out of an estimated population of 36 (72.2%), 12 came from *Dagobert*—situated a 2-h walk from *Sophie*—out of 37 estimated (32.4%), and five from *Repentir*—a 6-h walk from *Sophie*—out of 200 estimated (2.5%, four refusals) (Table 2). Two persons declared they had just arrived, and one was from another mining site.

**Table 2:** Characteristics of the study population

	Whole assessed population N = 46	Mining site			Others N = 3	p value
		Sophie N = 26	Dagobert N = 12	Repentir N = 5		
Sociodemographic data						
Population						
Estimated population in the mining site	na	36	37	200	na	
Proportion of population assessed	na	72.2%	32.4%	2.5%	na	
Sex						
Male	37 (80.4%)	23 (88.5%)	9 (75%)	4 (80%)	1 (33.3%)	0.137
Female	9 (19.6%)	3 (11.5%)	3 (25%)	1 (20%)	2 (66.7%)	
Place of birth						
Brazil	46 (100%)	26 (100%)	12 (100%)	5 (100%)	3 (100%)	0.042
Maranhão	17 (37%)	12	3	0	2	
Para	12 (26.1%)	6	6	0	0	
Amapa	4 (8.7%)	1	1	2	0	
Other states	12 (26.1%)	7	2	2	1	
Missing data	1 (2.2%)	0	0	1	0	
Time in gold mining (in months)						
Average (sd)	73 (± 94)	92 (± 85)	53 (± 62)	26 (± 52)	145 (± 248)	
Median [interquartile range]	48 [8–120]	78 [36–120]	30 [0.75–96]	5 [1–5]	3 [1–432]	
Medical data						
Malaria crises in the last 3 months (declared)						
At least one	21 (45.7%)	14	6	1	0	0.219
None	22 (47.8%)	11	5	3	3	
Missing data	3 (6.5%)	1	1	1	0	
Malaria attack rate in the last 3 months	na	53.8% [34.6–73]	50% [21.7–78.3]	20% [− 15.1 to 55.1]	na	
RDT						
Number of RDTs performed	13	7	4	1	1	
Number of RDTs positive	2	1 Pf	1 non-Pf	0	0	
PCR						
Number of PCR performed	43	25	11	5	2	0.403
Number of PCR positive	19	15	2	1	1	
Prevalence [IC95%]	44.2% [29.4–59]	60% [40.8–79.2]	18.2% [− 4.7 to 40.7]	^a^	^a^	
Asymptomatics carriers	15 (78.9%)	12 (80%)	1 (50%)	1 (100%)	1 (100%)	

^a^Not calculated because of sample size

The gender ratio was 4 (37 males÷9 females), the mean age was 36 years (SD = 10.8). All were Brazilian. They had been garimpeiros for a mean of 6.5 years (SD = 7.8), median = 4 years [0.6–10]. The average duration of onsite presence was 6 months (SD = 9), but 18 (39%) had arrived less than 3 months before, so were not yet present at the time of the outbreak signal. Places visited just before arriving were other illegal gold mines in French Guiana (20), Brazil (17), and Suriname (9). Six (13%) declared staying several years without leaving the forest (maximum = 6 years).

### Case detection

Eight patients presented fever, and one had cutaneous pallor. Hackett’s score was under two for all patients. Thirteen RDT were performed, diagnosing two malaria attacks (one *P. falciparum* from *Sophie* and one non-*P. falciparum* from *Dagobert*). Subsequently, 19 RT-PCR were positive for *P. vivax* among the 43 performed, which included the two persons with positive RDT. Twenty-one patients (21/46, 45.7%) declared having had at least one clinical malaria episode during the past 3 months, among which 12 were incapacitated for work (12/21, 57.1%).

Almost three quarters of *Sophie*’s population were evaluated. At this mining site, PCR-*Plasmodium* prevalence was estimated to be 60% (15/26) (95% CI 40.8–79.2), of which 80% were asymptomatic. The malaria attack rate was estimated at 53.8% (95% CI 34.6–73] in the past 3 months but should be interpreted carefully because of memory bias, extreme mobility and excessive diagnosis of malaria in case of fever.

### Indirect approach by questionnaire

Sixteen patients (16/46, 34.8%) reported that there had been the usual seasonal increase of malaria incidence during the rainy season, whereas nine (9/46, 19.6%) did not notice any increase. Nobody has reported severe malaria attacks or death around them. Participants declared that the malaria increase did not concern the *Sophie* mining site, but more the *Repentir* mining site, because of a larger population.

Overall, 30 patients declared a past history of malaria attack (30/46, 65.2%), among whom 19 used self-medication, all with artemether–lumefantrine (19/30, 63.3%). However, only one participant took the adequate dosage. According to 24 persons (24/46, 52.2%), malaria treatment had been more difficult to find on the black market at the gold mining sites for about 6 months, with fewer dealers present. Garimpeiros declared that Artecom^®^ (dihydroartemisinin–piperaquine–trimethoprim), the main drug used in self-medication in the region (15) was scarce, sometimes replaced by Coartem^®^ (artemether–lumefantrine). Thus self-medication was more difficult than it used to be. Participants declared they saved some anti-malarial tablets by interrupting treatment immediately after the end of symptoms, often as early as the second day.

Concerning prevention, only ten patients declared using a mosquito net (10/46, 21.7%). The reasons mentioned by the others were: not having any (21/31, 67.7%), not practical/not comfortable (6/31, 19.4%), burnt during military operations (4/31, 12.9%) (5 missing data).

Finally, 28 (28/46, 61%) said that there was an increase in people leaving *Sophie* mainly because of a lower profitability (unfavourable balance between gold production versus police destruction). They had moved to Suriname according to 18 persons (39%).

## Discussion

### Limitations of this investigation

This malaria outbreak investigation could not rely on classical malaria indicators (attack rate, incidence) because of the specific context: excessive presumptive diagnosis of malaria in case of fever without confirmation, mobility, memory bias and absence of historical data. Therefore, investigators decided to add unusual data sources, such as qualitative questionnaire and RT-PCR on blotting paper.

According to garimpeiros, the mining site called “*Sophia*” in Brazilian actually comprises three different mining sites called in French “*Sophie*”, “*Repentir*” and “*Dagobert*” close to each other. Thus, the signal coming from Suriname reporting that there were patients coming from *Sophia* probably concerned these three sites. This emphasizes the importance of clearly defining terms and names, in particular in this context of multiple languages (Brazilian for garimpeiros, French in French Guiana, English and Dutch in Suriname). Thus this investigation may not reflect the exact situation of malaria at the whole “*Sophia*” mining site.

### Conclusions of the investigation

This investigation led us to the following conclusions:

1. The increase of imported malaria cases in Suriname was presumed to result from the increase in gold miners’ mobility and from the decreased availability of under-the-counter anti-malarial treatment, which may have caused people to move to Suriname to seek care.
  According to persons met during the investigation, garimpeiros mobility to Suriname increased in early 2017 because of an unfavourable balance between gold production and police destruction in this area. Garimpeiros would leave the production areas and stay a little longer in Suriname waiting to decide where to go next. Interviewed persons declared that it had been more difficult to find under-the-counter anti-malarial treatment for about 6 months. The fragmentation of sites by operations of the French Armed Forces may have decreased the profitability of trade, especially for products considered non-essential, such as anti-malarial treatment. This could have explained the decreased availability of anti-malarial drugs at gold mining sites. The poor treatment adherence reported here is concordant with what has been observed in other studies in gold miner populations, and it confirms the threat of emergence of artemisinin resistant *Plasmodium* on the Guiana Shield, as observed in South-East Asia [4, 8, 15–17]. Although there is a crisis in Venezuela fueling mass emigrations, there have been no reports of Venezuelan migrants so far. Given the burden of malaria in Venezuela this may have epidemiological consequences for malaria in the region.
2. The investigation could not conclude to a malaria outbreak in *Sophie* at the time of the investigation. It could be because of the delay between the signal, its retransmission to RHA and the field mission. Passive case detection during the mission diagnosed one single clinical malaria case in *Sophie* (2 in the whole study population), whereas the RT-PCR prevalence was very high (60%). This suggested an endemic situation with repeated contact with *Plasmodium* conducting to partial immunity, thus carriage of *Plasmodium* with low parasitaemia, not detectable with RDTs. These results are in concordance with previous studies assessing malaria on illegal gold mining sites in French Guiana: the first one on the Maroni river banks in Suriname in 2015 found a malaria prevalence of 46.4% among garimpeiros coming from *Sophia* 3); the second one on a nearby site named *Eau Claire* in 2014 revealed a prevalence of 48.3% [4].
3. Participants declared that there was a malaria increase in *Repentir* mining site. This information could not be verified on the field because French Armed Forces were conducting a police operation in *Repentir* at the time of the malaria investigation mission. According to participants, this increase was thought to be linked to the rainy season (January to June) as usually reported by the French Malaria surveillance system in this region [18], which would lead to the same situation every year, and to a greater number of people working there (about 200 garimpeiros).

### A surprising predominance of *P. vivax*

One patient had a positive RDT for *P. falciparum* with positive RT-PCR for *P. vivax*. This discordance could be explained by low *P. vivax* parasitaemia that was detected by RT-PCR, but not by RDT, associated with a prior *P. falciparum* attack diagnosed and treated 20 days before. Indeed, the HRP2 antigen can be present for 2 to 3 weeks after treatment. However, it was surprising to find only *P. vivax* in RT-PCR positive samples because both former studies highlighted that *P. falciparum* was predominant in garimpeiros [2, 3]. The epidemiology of malaria in remote illegal gold mines in FG is not well described and it may be explained by different vector distributions and competences, leading to different hotspots where transmission is maintained by different species [13]. The proximity of gold mining sites to populations more or less refractory to *vivax* (Maroon populations are largely Duffy negative) could maybe also have an effect.

### Response to the situation

This investigation reveals a lack of access to diagnosis and treatment in the garimpeiros population and emphasizes the potential challenges for the Guiana Shield of the absence of malaria control in this population, such as the increase of malaria cases in neighboring countries. As a malaria outbreak was not confirmed in *Sophie*, lobbying to promote malaria control in this specific population was done rather than setting up a specific action. Actually access to free-of-charge diagnosis and treatment is theoretically possible for garimpeiros in FG but health centres are far away from gold mining camps. The journey to the health centre is very expensive, making some prefer self-medication [15]. The lack of knowledge of proper dosages and the high cost of under-the-counter treatment lead to poor adherence. French regulations do not allow non-physician health workers to deliver treatment, therefore, control programmes such as the one in neighbouring Suriname, where community health workers are employed, cannot be implemented [6]. Dealing with geographical and regulatory issues in this very particular context, a new strategy called “Malakit” is currently tested as a pilot project in collaboration with Suriname and Brazil [19], mostly funded by European Regional Development Funds, Regional Health Agency of French Guiana, Global Fund and Pan-American Health Organization. This strategy is based on the distribution of kit for malaria self-diagnosis and self-treatment, the “malakit”, each containing three Rapid Diagnostic Tests and one complete treatment with ACT and a single dose of primaquine, after training by health facilitators. The distribution sites are spread along the borders with Brazil and Suriname, were gold miners come for rest or logistical supplies [19]. It presently seems the only way to control malaria among garimpeiros in FG.

Vector control should also be implemented. Despite poor knowledge of malaria vectors in mining camps in FG, vector control should include mosquito net distribution for gold miners. However, European legislation currently complicates the use of insecticide-impregnated bed nets. Moreover, during police operations, equipment in illegal mines is destroyed, including mosquito nets.

## Conclusion

This investigation of a suspicion of a malaria epidemic used unusual tools due to particular remoteness of studied area and logistical constraints, in the image of Rapid Epidemiological Assessment Methods [20]. Even if part of the assessment was based on declarations from gold miners themselves, the biological results confirms the importance of malaria among garimpeiros as already demonstrated in the literature [5, 13]. Their mobility along the Guiana Shield and their health-seeking behaviour are likely to spread malaria in new populations and derail malaria elimination efforts that are underway [6]. In this hard to reach context, implementation of malaria control measures is thus challenging but urgently needed. Malakit pilot project actually in progress could be a solution.

## Abbreviations

AMC: asymptomatic malaria carriage
FG: French Guiana
RHA: Regional Health Agency
RDT: rapid diagnostic test
